# Minor Surgical Gynecology

**Published:** 1885-06

**Authors:** 


MINOR SURGICAL GYNECOLOGY; A Treatise of Uterine Diagnosis and the Lesser Technicalities of Gynecological Practice, including General Rules for Gynecological Operations and the Operations for Lacerated Cervix and Perineum, and Prolapsus of Uterus and Vagina, for the use of the advanced student and general practitioner. By Paul F. Mundé, M. D., Professor of Gynecology at the N. Y. Polyclinic and at Dartmouth College; Gynecologist to Mt. Sinai Hospital; Obstetric Surgeon to Maternity Hospital; V. P. of the American Gynecological Society; Fellow of the Obstetrical Society of New York; Corresponding Fellow of the Obstetrical Societies of Edinburgh and Philadelphia and of the Gynecological Society of Boston; Honorary Fellow of the Medical and Surgical Society of Richmond, Va., of the Pathological Society of Harrisburg, Penn., and of the Medical Society of the County of Fairfield, Conn. Second edition, revised and enlarged, with three hundred and twenty-one illustrations. New York: William Wood & Co., 56 and 58 Lafayette Place. 1885.

It is evident, from the prominent attitude of those members of the profession who have devoted themselves to the study and treatment of the diseases pertaining to woman, and from the rapid accumulation of facts and the dissemination of knowledge respecting female diseases, that there must exist at present peculiar tendencies to derangement of the female organization. This is preëminently a gynecological era, in which there is a demand for a treatise of uterine diagnosis and the lesser technicalities of gynecological practice, including general rules for gynecological operations, and the operations for lacerated cervix and perineum, and prolapsus of uterus and vagina, such as Dr. Mundé has presented to the medical public in this volume of 552 pages. We learn, however, from the preface, that not the half has been told of the wonders of operative gynecology, as the author has only chosen for the present several minor operations which are now agitating the professional mind more particularly, and which bid fair to become more universally popular, leaving for a future time the preparation of a more complete work.

This accounts, doubtless, for the omission, on this occasion, of any notice of the important services and valuable practical additions to gynecology by one of his co-laborers in this department in the city of New York; and it is fair to infer that the keen discernment of a progressive utilitarian spirit, such as is supposed to actuate a teacher of others, shall not in future overlook the advances and improvements recognized throughout the medical world.

If we are to judge of the exhaustive consideration of details to be undertaken at a future day, from the minuteness of the description now presented of the mode of conducting the examination of a female patient, it would seem that the author presumes largely upon the want of common sense, not to say professional knowledge, on the part of the advanced student and general practitioner for whom his instructions are intended.

After a general survey of the abdominal regions, indications are presented for detecting everything, from crab lice outside the vulva to a child within the womb, and to tumors in the abdominal cavity. The wood cuts of instruments and their mode of application are instructive, and the delineation of displacements of the womb, with their adjustment by tamponage and pessaries, are useful illustrations.

The vexed question of dilatation of the cervical canal by the various agencies of tents and pliable or solid dilators receives a large share of attention, with the conclusion that "whenever thorough, rapid and safe dilatation by gradual methods is desired, the tupelo tent is the agent to be chosen," and "when rapid dilatation of an already dilated canal is intended, the inflatable rubber tubes of Molesworth are the best instruments." Again, "the degree of constriction of the cervical canal, which would call for enlargement by the knife, is largely relative, or is a matter of opinion as to the absolute necessity of a patulous canal for the possibility of conception." "In this country the metrotomes with hidden blades are little used, but the knife introduced by Sims is almost universally employed." "The canal having been thoroughly dilated, the cervix is seized with the tenaculum drawn down and a smooth glass or hard rubber plug is introduced to arrest hemorrhage and maintain the canal patent." In treating of the operation for laceration of the cervix uteri, it is stated that Emmet claims that general anemia from defective innervation of the nutrient organs is one of the results of reflex neurosis from a lacerated cervix. He is positive in this statement, although most gynecologists have not yet accepted his view in all its bearings. Dr. Mundé claims that the significance of a cervical rent as a cause of uterine diseases lies not in the existence of the rent itself, but solely in the symptoms which it produces, and in the direct influence which can be traced to it as the prime factor in the production or maintenance of some pathological condition or functional derangement in the pelvic organs or elsewhere in the body.

The radical treatment of a lacerated cervix, with or without complications, consists solely in paring the edges of the rent and approximating them by sutures until nature has united the raw surfaces permanently. Details of this operation, with its risks and possible evil results, are set off against the possible results if it is not performed.

The volume closes with the operations for lacerated perineum, rectocele, cystocele, and prolapsus of the uterus and vagina.

Ovariotomy, with Battey's and Tate's operations, are only referred to cursorily, and ablations of the womb with Cesarian section are left for the specialist, though more likely to require the attention of the general practitioner than many of the matters presented. The extended sphere of observation in cases of prolapsed ovaries, which a report made by Dr. Mundé some years ago indicated, would have warranted something more than the brief paragraph, stating that the replacement of the prolapsed ovaries by the manual and partial methods described for retroversion of the uterus, is easily effected, unless they are adherent.

While more is said in treating of some points than is called for by the nature of the case, other matters of paramount importance are passed over with slight notice; yet, upon the whole, this second edition is an improvement upon the first, and gives much practical instruction for the student.

